# Evaluation of Multi-Frequency Bioelectrical Impedance Analysis against Dual-Energy X-ray Absorptiometry for Estimation of Low Muscle Mass in Older Hospitalized Patients

**DOI:** 10.3390/jcm13010196

**Published:** 2023-12-29

**Authors:** Rikke Lundsgaard Nielsen, Aino Leegaard Andersen, Thomas Kallemose, Morten Damgaard, Olivia Bornæs, Helle Gybel Juul-Larsen, Louise Westberg Strejby Christensen, Baker Nawfal Jawad, Ove Andersen, Henrik Højgaard Rasmussen, Tina Munk, Trine Meldgaard Lund, Morten Baltzer Houlind

**Affiliations:** 1 Department of Clinical Research, Acute CAG, Copenhagen University Hospital Amager and Hvidovre, 2650 Hvidovre, Denmark; aino.leegaard.andersen@regionh.dk (A.L.A.); thomas.kallemose@regionh.dk (T.K.); olivia.bornaes@regionh.dk (O.B.);; 2 Department of Clinical Medicine, Faculty of Health and Medical Sciences, University of Copenhagen, 2100 Copenhagen, Denmark; 3 Department of Clinical Physiology and Nuclear Medicine, Centre for Functional and Diagnostic Imaging and Research, Copenhagen University Hospital Amager and Hvidovre, 2650 Hvidovre, Denmark; 4 The Capital Region Pharmacy, Marielundsvej 25, 2730 Herlev, Denmark; 5 Emergency Department, Copenhagen University Hospital Amager and Hvidovre, 2650 Hvidovre, Denmark; 6 Center for Nutrition and Intestinal Failure, Aalborg University Hospital, Aalborg University, 9220 Aalborg, Denmark; hhr@rn.dk; 7 The Dietitians and Nutritional Research Unit, EATEN, Copenhagen University Hospital—Herlev and Gentofte, 2100 Copenhagen, Denmark; 8 Department of Drug Design and Pharmacology, University of Copenhagen, 2100 Copenhagen, Denmark; trine.lund@sund.ku.dk

**Keywords:** older patients, malnutrition, sarcopenia, muscle mass, BIA, DXA

## Abstract

The accuracy of multi-frequency (MF) bioelectrical impedance analysis (BIA) to estimate low muscle mass in older hospitalized patients remains unclear. This study aimed to describe the ability of MF-BIA to identify low muscle mass as proposed by The Global Leadership Initiative on Malnutrition (GLIM) and The European Working Group on Sarcopenia in Older People (EWGSOP-2) and examine the association between muscle mass, dehydration, malnutrition, and poor appetite in older hospitalized patients. In this prospective exploratory cohort study, low muscle mass was estimated with MF-BIA against dual-energy X-ray absorptiometry (DXA) in 42 older hospitalized adults (≥65 years). The primary variable for muscle mass was appendicular skeletal muscle mass (ASM), and secondary variables were appendicular skeletal muscle mass index (ASMI) and fat-free mass index (FFMI). Cut-off values for low muscle mass were based on recommendations by GLIM and EWGSOP-2. MF-BIA was evaluated against DXA on the ability to estimate absolute values of muscle mass by mean bias, limits of agreement (LOA), and accuracy (5% and 10% levels). Agreement between MF-BIA and DXA to identify low muscle mass was evaluated with sensitivity, specificity, negative predictive value (NPV), and positive predictive value (PPV). The association between muscle mass, dehydration, malnutrition, and poor appetite was visually examined with boxplots. MF-BIA overestimated absolute values of ASM with a mean bias of 0.63 kg (CI: −0.20:1.46, LOA: −4.61:5.87). Agreement between MF-BIA and DXA measures of ASM showed a sensitivity of 86%, specificity of 94%, PPV of 75% and NPV of 97%. Boxplots indicate that ASM is lower in patients with malnutrition. This was not observed in patients with poor appetite. We observed a tendency toward higher ASM in patients with dehydration. Estimation of absolute ASM values with MF-BIA should be interpreted with caution, but MF-BIA might identify low muscle mass in older hospitalized patients.

## 1. Introduction

Malnutrition is defined as “a state resulting from lack of intake or uptake of nutrition that leads to altered body composition (decreased fat-free mass (FFM)) and body cell mass leading to diminished physical and mental function and impaired clinical outcome from disease” [1]. Sarcopenia is present when low muscle strength and low muscle quantity or quality are detected [2].

Older patients with malnutrition and sarcopenia often have skeletal muscle wasting, which is associated with serious outcomes such as loss of independence, reduced quality of life, increased morbidity, and mortality [2,3]. Older adults admitted to an emergency department (ED) represent a risk population in relation to malnutrition and sarcopenia [4,5]. Patients admitted to an ED are often discharged without being transferred to another department, and due to the short stays, some patients will be discharged before interventions to treat malnutrition and sarcopenia have been initiated [6,7,8]. Correct identification of malnutrition and sarcopenia in the ED is therefore of great clinical value because the conditions largely can be treated by adequate nutritional and exercise interventions [9]. Various criteria to identify malnutrition and sarcopenia have been proposed. Recently, The Global Leadership Initiative on Malnutrition (GLIM) has published a set of diagnostic criteria for the diagnosis of malnutrition and recommends the assessment of muscle mass [3]. The European Working Group on Sarcopenia in Older People (EWGSOP-2) has recommended that reduced muscle strength should be confirmed by evaluation of low muscle mass for the diagnosis of sarcopenia [2]. Thus, malnutrition and sarcopenia diagnosis based on GLIM and EWGSOP-2 require accurate methodologies to detect low muscle mass. As proposed by GLIM and EWGSOP-2, low muscle mass can be detected via, e.g., appendicular skeletal muscle mass (ASM), appendicular skeletal muscle mass index (ASMI), or fat-free mass index (FFMI) [2,3]. Two techniques are commonly used to estimate muscle mass: dual-energy X-ray absorptiometry (DXA) and bioelectrical impedance analysis (BIA) [10]. To date, DXA appears more accurate for estimating muscle mass, and the technique has gained acceptance in clinical research and is endorsed by the GLIM and EWGSOP-2 consortium, but it is not feasible in a clinical setting such as an ED due to short stays and required financial and personal resources [2,10]. DXA works by sending dual low-dose X-ray beams with different energy levels through the body, which can differentiate between bone mineral, lean mass, and fat mass [11]. BIA is also endorsed as a method to estimate muscle mass and appears more feasible in an ED than DXA as it can be performed bedside and does not require special training [2,10]. BIA is conceptually based on the electrically conductive properties of the human body and is performed with single-, dual-, or multi-frequency (MF) technology. Measures of bioelectrical conductivity are proportional to total body water and the body compartments with high water contents, such as fat-free mass and skeletal muscle mass [11]. Body composition is either estimated on whole-body impedance (no torso measurement) with empirical equations based on factors like age and sex or direct segmental measurements without using empirical data [11].

The use of muscle mass estimates to diagnose malnutrition and sarcopenia is sparsely implemented in older adults, primarily due to uncertainty regarding which method to use to measure muscle mass. A review by Cederholm et al. summarizes 14 publications that have applied GLIM in older populations and finds that the estimation of muscle mass is missing in most papers [12]. A review by Barazzoni et al. concludes that the methodological issues in estimating muscle mass have been a limiting factor in the implementation of the EWGSOP-2 sarcopenia diagnostic approach [13]. Recent research shows suitable agreement between MF-BIA and DXA to measure the absolute values of FFM in younger healthy individuals [14]. But, this was not seen in a study using dual-frequency (DF)-BIA in older adults [15]. Only a limited number of studies have compared the performance of MF-BIA against DXA to estimate absolute values of muscle mass in the older population, and research on the ability of MF-BIA to identify low muscle mass in the older population is even more scarce [16,17,18,19,20]. Therefore, this study aims to evaluate MF-BIA against DXA for the direct estimation of low muscle mass in older hospitalized patients. Secondly, the study aims to assess the association between muscle mass, dehydration, poor appetite, and malnutrition. 

## 2. Materials and Methods

### 2.1. Study Design and Setting

In this protocolized prospective exploratory cohort study, we examined data from a subpopulation of the randomized clinical trial, OptiNAM, investigating the effect of optimizing nutrition and medication in acutely admitted older patients registered at Clinical Trials.gov (NTC03741283). A detailed description of the study design can be found elsewhere [21,22]. Patients were recruited at the ED at Copenhagen University Hospital, Hvidovre, Denmark. The ED annually handles 12,600 acute admissions. Patients enrolled in the original study were additionally invited to a sub-study where they received a glomerular filtration rate measurement (mGFR) and an estimation of muscle mass based on MF-BIA and DXA measurements. This study is based on data from the above-mentioned sub-study. The reporting of this study follows the “Strengthening The Reporting of Observational studies in Epidemiology (STROBE)” guidelines [23].

### 2.2. Patients and Recruitment

Eligibility was identified through the electronic patient journal. Patients were eligible for the original study if they were ≥65 years of age, acutely admitted to the ED at Copenhagen University Hospital, Hvidovre, Denmark, community-dwelling, and residing in districts of West or Southwestern Copenhagen.

Patients were excluded from the original study if they were unable to understand Danish, cooperate physically (e.g., hearing or speech impairment) or cognitively (e.g., dementia or unconsciousness), were in an isolation room, were not Caucasian, or were admitted due to suicide attempt or terminal illness. Additional exclusion criteria for this study were prior amputation, ascites, lower extremity edema, and pacemaker on the days of MF-BIA and DXA measurements (hereafter referred to as test days). Patients were informed that they could decline and withdraw from this sub-study for any reason and that this would not have any consequences for their regular course of treatment. In this current study, older hospitalized patients are considered to be patients who have been admitted to the ED and then either directly discharged from the ED or transferred to another department, as previously described in detail [22].

### 2.3. Variables and Data Collection

Data were collected by trained study staff at two time points, in the ED directly after inclusion in the original study and on test days. Data were entered directly in the electronic patient records or in electronic case report forms (CRF) in REDCap (Research Electronic Data Capture, Vanderbilt University, Nashville, TN, USA). If not directly entered, paper CRFs were double entered and validated in REDCap.

#### 2.3.1. Patient Demographics

Patient characteristics were based on self-report measurements by study staff or from the medical records and included sex, age, weight, height, admission diagnoses, length of stay (LOS), readmissions (within 30 days), and mortality (3 months and 1 year). Admissions diagnoses were determined by the International Classification of Diseases (ICD-10) diagnosis codes. The Charlson Comorbidity Index (CCI) was used to quantify disease burden and calculated without age correction [24]. 

#### 2.3.2. Muscle Mass Estimations 

MF-BIA and DXA technology were used to estimate muscle mass and were performed at the Department of Clinical Physiology and Nuclear Medicine, Centre for Functional and Diagnostic Imaging and Research, Copenhagen University Hospital, Hvidovre, Denmark. If possible, the estimations were performed during hospitalization (after study recruitment). Otherwise, patients were asked to return for estimations as soon as possible. Estimations were performed at the same time of the day and approx. three hours after breakfast. Patients were asked to refrain from strenuous physical activity, not to change their eating habits, and to drink water as usual on the day of the measurement. There was no systematic bladder voiding. Patients wore their own clothes without shoes, and all metal parts, such as watches and jewelry, were removed. While MF-BIA and DXA scans were performed, patients were asked to lay still. Age, height, and weight were recorded on test days and directly entered into the MF-BIA and DXA devices. Muscle mass variables, ASM (primary), ASMI, FFMI (secondary), and cut-off values were defined according to the most recent guidelines launched by GLIM and EWGSOP-2 (Table 1).

As a reference method, a whole-body DXA scan (Version 16) was performed before MF-BIA measurement (GE Lunar Prodigy Primo, GE Healthcare Technologies, Madison, WI, USA). Patients were positioned on the scanner table in a supine position with straight legs, feet held together with a Velcro band, and arms close to the body. The software automatically defined the region of the trunk and appendages, which were then adjusted manually, and then DXA software (Version 16) calculated whole- and regional body composition estimates. The same scanner was used for all measurements, and calibration was performed throughout the study according to local guidelines [25]. Scans were performed by designated and trained staff three hours after administration of the radioisotope used for the mGFR. Each scan had a mean length of five minutes. 

Whole-body MF-BIA was performed immediately after DXA measurement with a portable bedside direct segmental MF-BIA analyzer (InBody S10; Biospace, Seoul, Republic of Korea), which measures with six different frequencies (1, 5, 50, 250, 500, and 1000 kHz) at each of five segments (right arm, left arm, trunk, right leg, and left leg). Patients were placed on a hospital bed for approx. five minutes in a supine position with their arms not touching the trunk part of their body (approx. 15-degree angle) and legs in shoulder width position. Contact points on fingers and ankles were cleaned before touch-type electrodes were placed as described in the manufacturer’s standard protocol. Cases of impedance reverse were checked and registered if it was not possible to remove. Measurements lasted for 90 s.

#### 2.3.3. Hydration Assessment (Osmolarity and Medication Review)

Hydration was investigated by calculated plasma osmolarity and a pharmacist-led medication review.

Osmolarity was calculated using the ESPEN recommended equation: 1.86 × (Na^+^ + K^+^) + 1.15 × glucose + urea + 14. Cut-off: >295 mOsm/L [9]. Plasma isolated from whole blood was collected on test days prior to muscle mass estimations. P-Na^+^, P-K^+^, P-glucose, and P-urea were analyzed according to standard methods at the Department of Clinical Biochemistry, Copenhagen University Hospital, Rigshospitalet, Denmark. All measures were quantified in mmol/L.

A list of medications thought to potentially affect hydration was created by two pharmacists based on therapeutic indications and well-known side effects (such as edema) with inspiration from Walther et al. [26]. The following medication classes were considered: dehydration: loop-diuretics, thiazides, potassium-sparing diuretics, and renin–angiotensin–aldosterone system (RAAS) inhibitors; overhydration: calcium antagonists, alpha- and beta-blockers, nonsteroidal anti-inflammatory drugs (NSAIDs), glucocorticoids, and urinary tract agents. A pharmacist-led medication review was performed retrospectively in the electronic patient records by evaluating medication use on test days according to the list. The list of prescribed medications belonging to the selected medication classes can be found in the Supplementary Materials Table S1.

#### 2.3.4. Nutritional Screening 

Nutritional screening was performed at inclusion with the Mini Nutritional Assessment Short-Form (MNA^®^-SF). MNA^®^-SF is a validated screening tool for older adults in hospitals consisting of six questions regarding food intake, weight loss, mobility, psychological stress/acute disease, neuropsychological problems, and BMI [27]. Nutritional status was defined as normal nutritional status: score = 12–14; risk of malnutrition: score = 8–11; malnutrition: score = 0–7.

#### 2.3.5. Appetite Assessment

Appetite was assessed at inclusion using the screening tool Simplified Nutritional Appetite Questionnaire (SNAQ). SNAQ consists of four questions concerning appetite, fullness, taste, and daily meal frequency [28]. Poor appetite was defined as a SNAQ score ≤ 14. 

### 2.4. Statistical Methods

Categorical variables are expressed as frequencies and percentages. Continuous variables are expressed as mean and standard deviation (SD) or median and interquartile range (IQR) for normally and non-normally distributed data, respectively. Normality assumptions were evaluated by quantile–quantile plots (QQ plots). Scatterplots were used to visualize associations between MF-BIA and DXA for absolute values of ASM, ASMI, and FFMI. Bland–Altman analyses were conducted to evaluate bias, mean difference with 95% confidence intervals (CI), and limits of agreement (LOA) between absolute values of MF-BIA and DXA estimates. Additionally, the percentage of patients with accurate predictions within 5% and 10% were estimated. The ability of MF-BIA to identify low muscle mass according to DXA was estimated by sensitivity, specificity, positive predictive value (PPV), negative predictive value (NPV), agreement, and Cohen’s Kappa. All estimates are presented with 95% CI. Additionally, visual representations of the MF-BIA and DXA muscle mass distribution within dehydration, malnutrition, and poor appetite are presented as boxplots. Given that this is an exploratory sub-study, there is no separate sample size calculation. Data were analyzed using R version 4.2.2 [29].

## 3. Results

### 3.1. Patient Characteristics

**Figure 1:**
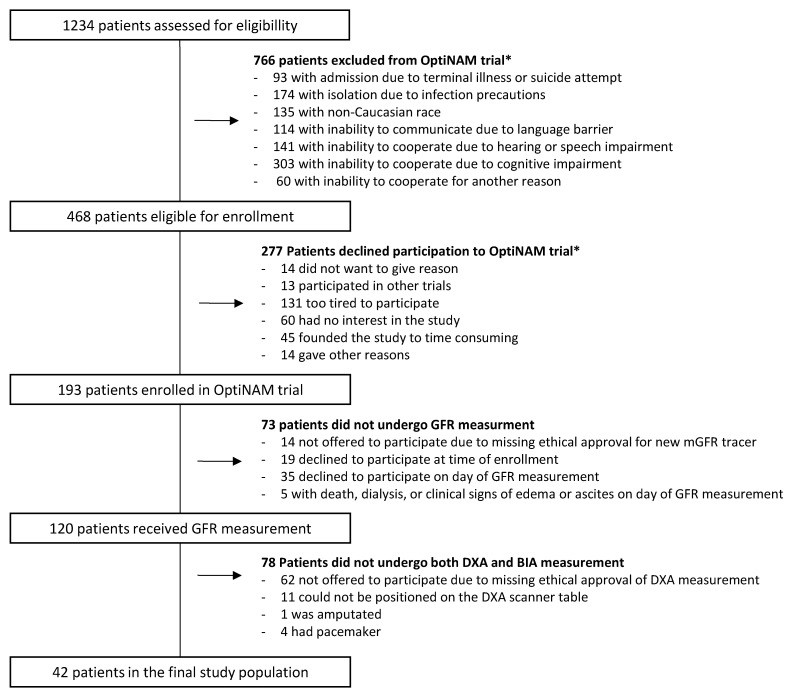
Flowchart of patients included in the study * More than one reason can exist.

In total, 193 patients were recruited to the original study from October 2018 to April 2021. Of the 193 patients, 120 patients underwent GFR measurement of which 78 patients did not have both MF-BIA and DXA measurements. Hence, 42 patients were included in this study (Figure 1). Patient characteristics are shown in Table 2, and details regarding medication related to hydration can be found in the Supplementary Materials Table S2. The distribution of mean values for BMI (26.3 vs. 27), age (79.2 vs. 78.9), and sex (females: 58.5 vs. 64.3) in this sub-study was comparable to that of the main study. In total, 64.3% of patients had their MF-BIA and DXA measurements performed within 2 weeks after discharge.

### 3.2. The Evaluation of MF-BIA against DXA to Estimate Absolute Values of Muscle Mass

**Figure 2:**
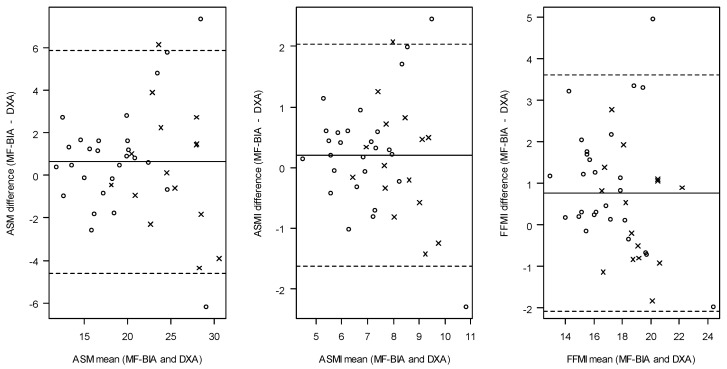
Bland–Altmann plots for agreement between ASM, ASMI, and FFMI estimated by MF-BIA and DXA (absolute values). Dots show females, and crosses show males. Dashed lines represent the limits of agreement, and plain lines represent the mean bias. Abbreviations: MF-BIA: multi-frequency bioelectrical impedance analysis, DXA: dual-energy X-ray absorptiometry, ASM: appendicular skeletal muscle mass, ASMI: appendicular skeletal muscle mass index, FFMI: fat-free mass index.

**Figure 3:**
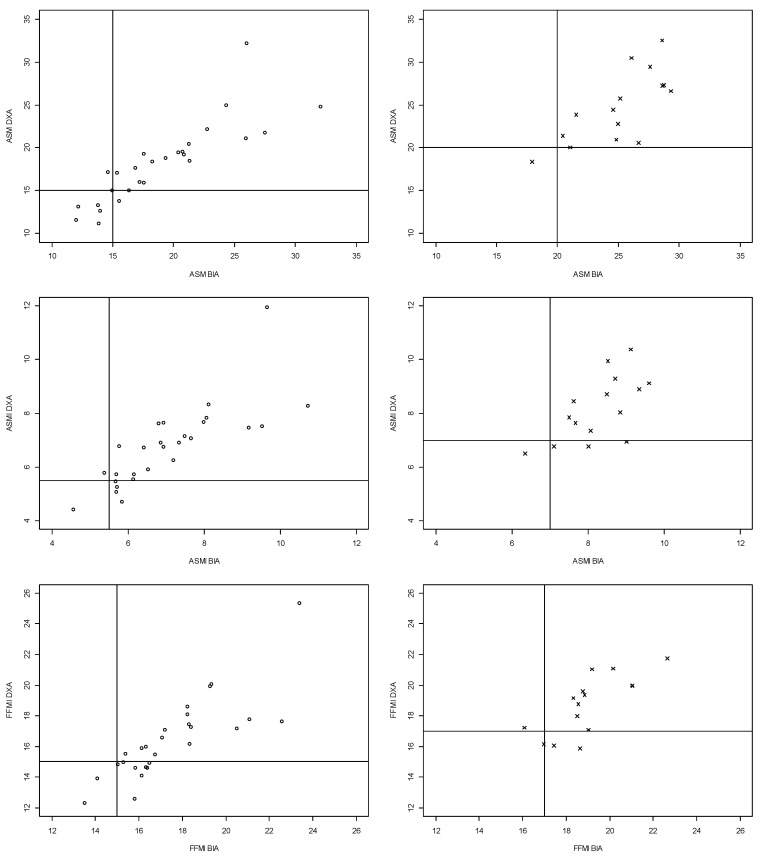
Scatter plots for agreement between ASM, ASMI, and FFMI estimated by MF-BIA and DXA (absolute values and low muscle mass). Dots show females, and crosses show males. Solid lines represent the cut-off values for low muscle mass. Abbreviations: ASM: appendicular skeletal muscle mass, DXA: dual-energy X-ray absorptiometry, MF-BIA: multi-frequency bioelectrical impedance analysis, ASMI: appendicular skeletal muscle mass index, FFMI: fat-free mass index.

The ability of MF-BIA to estimate absolute values of ASM, ASMI, and FFMI is presented in Table 3 and Table 4. Bland–Altman plots and Scatterplots (Figure 2 and Figure 3) visualize the agreement between MF-BIA and DXA for absolute values of ASM, ASMI, and FFMI. In Table 3, the distribution of absolute mean values for muscle mass based on ASM, ASMI, and FFMI appeared similar for MF-BIA and DXA, whereas this was not the case for the distribution of low and normal muscle mass based on ASMI and FFMI. Agreement between absolute values of ASM, ASMI, and FFMI estimated by MF-BIA and DXA as investigated with Bland–Altman showed that MF-BIA systematically overestimated ASM, ASMI, and FFMI compared to DXA with a mean bias of 0.63 kg (−0.20:1.46), 0.21 kg/m^2^ (−0.08:0.50) and 0.76 kg/m^2^ (0.31:1.22), respectively. The lower and upper LOAs were as follows for ASM: −4.61 kg to 5.87 kg; ASMI: −1.62 kg/m^2^ to 2.04 kg/m^2^; FFMI: −2.08 kg/m^2^ to 3.61 kg/m^2^. The percentages of accurate MF-BIA estimates within 10% of DXA values for absolute ASM, ASMI, and FFMI were between 62 and 74%.

### 3.3. Agreement between MF-BIA and DXA to Estimate Low Muscle Mass

In Figure 3, discrepancies between MF-BIA and DXA measures of ASM, ASMI, and FFMI are visualized. Calculating the number of patients with an ASM value of a maximum of 1.5 kg more than current ASM cut-off values showed that three female patients were deemed to have normal muscle mass due to ASM estimates of 15.06 kg, 15.93 kg, and 16.02 kg, respectively, and one male patient due to an ASM estimate of 20.03 kg. The corresponding values for sensitivity, specificity, NPV, PPV, kappa, and overall agreement can be found in Table 4. Sensitivity for detecting low ASM, ASMI, and FFMI using MF-BIA were 86%, 22%, and 23%, respectively. Further, the PPV for ASM, ASMI, and FFMI were 75%, 67%, and 75%, respectively.

### 3.4. Associations between Absolute Values of Muscle Mass with Dehydration, Malnutrition, and Poor Appetite

**Figure 4:**
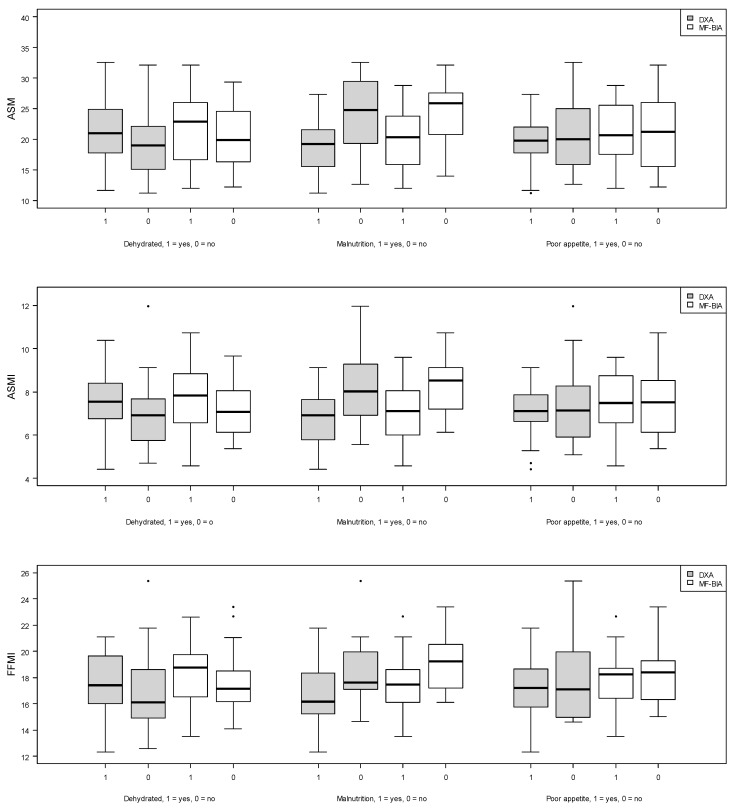
Boxplots for associations between absolute values of muscle mass with dehydration, malnutrition, and poor appetite. Gray represents DXA measures, and white represents MF-BIA measures. Abbreviations: ASM: appendicular skeletal muscle mass, DXA: dual-energy X-ray absorptiometry, MF-BIA: multi-frequency bioelectrical impedance analysis, ASMI: appendicular skeletal muscle mass index, FFMI: fat-free mass index.

Figure 4 indicates that patients with dehydration had higher median values of ASM, ASMI, and FFMI, as measured by MF-BIA and DXA. Further, patients with malnutrition appeared to have lower median values of ASM, ASMI, and FFMI, as estimated by both MF-BIA and DXA (Figure 4). Lastly, median values of ASM, ASMI, and FFMI for both MF-BIA and DXA appeared to be similar regardless of normal or poor appetite (Figure 4).

## 4. Discussion

### 4.1. Main Findings

In our study, we investigated the accuracy of MF-BIA to estimate low muscle mass following recommendations by GLIM and EWGSOP-2 in older hospitalized patients. We observed that MF-BIA overestimated absolute values of ASM, ASMI, and FFMI compared to DXA in this study sample. However, based on the CI values, the degree of bias could not be determined for ASM and ASMI, whereas the CI values for FFMI indicate clear bias. ASM estimated by MF-BIA was superior in detecting low muscle mass compared to ASMI and FFMI when evaluated against DXA. Additionally, patients with dehydration appeared to have higher median values of ASM, ASMI, and FFMI compared to patients without dehydration. We observed lower median values of ASM, ASMI, and FFMI in malnourished patients compared to well-nourished patients. This was not the case for patients with poor appetite compared to patients with normal appetite.

### 4.2. Comparison to Other Studies

The present study showed bias and wide CI values for muscle mass estimated with MF-BIA against DXA. This indicates that the individual differences between muscle mass estimated by MF-BIA and DXA are subject to great variability, and thereby, it may be an issue when MF-BIA is used to assess muscle mass. No studies were comparable to ours regarding the device used and our patient population. Similar to our findings, two studies have reported wide LOAs for different body composition variables when DF-BIA and MF-BIA were compared with DXA in older patients [15,16]. Contradicting our results, Jayanama et al. found that InbodyS10, similar to our device, had high correlations of FFMI compared with DXA in maintenance hemodialysis Thai patients [17]. This inconsistency in our findings could be explained by ethnic differences in body composition [30]. In line with our results, Buckinx et al. reported poor agreement between InBodyS10 and DXA in a subgroup analysis of healthy older patients [18]. The degree of accuracy in estimating muscle mass with BIA in older adults is inconsistent both in terms of methods and results. Therefore, it is difficult to evaluate the clinical implication of the differences in the muscle mass estimates between the studies. Research dedicated to improving the techniques for estimating muscle mass in clinical practice appears highly relevant.

To the best of our knowledge, few studies have dealt with the ability of MF-BIA against DXA to identify low muscle mass as proposed by GLIM and EWGSOP-2 in older adults [19,20,31,32,33]. We found a reasonable sensitivity and specificity for ASM, and current results mostly support our findings. Three studies have found reasonable sensitivity and specificity to identify older adults with low ASM using different frequency technology and predictive equations (MF-BIA, single-frequency (SF)-BIA, and SF-BIA with three different equations) [19,31,33]. In contrast, two studies have reported that SF-BIA and MF-BIA (InbodyS10) misclassified older adults as non-sarcopenic compared to DXA [20,32]. The discrepancies between these findings and our findings may be attributed to differences in the characteristics of the study sample and the technical specifications of the BIA devices. This addresses a significant shortfall in the diagnosis of malnutrition and sarcopenia. The use of BIA may be a suitable substitute for DXA to identify trends for muscle mass in clinical practice, taking its feasibility into consideration. However, a thorough clinical evaluation should be performed if low muscle mass is suspected. To better understand MF-BIA performance, we are assessing muscle mass using both MF-BIA and DXA in our ongoing and upcoming studies with older hospitalized patients [34]

We assessed edema as a physical examination and calculated plasma osmolarity as recommended by ESPEN. Munk et al. have previously shown that the osmolarity equation recommended by ESPEN accurately determines water-loss dehydration in older hospitalized patients [35]. According to the ESPEN osmolarity equation, 32% of our study sample was dehydrated. A similar prevalence of 37% was found in a study with older adults admitted to an ED, although their study was based on measured osmolarity (cut-off >300 mOsm/kg) [36]. 

Contrary to common assumptions, we observed that patients being dehydrated had higher muscle mass estimates [10]. These results must be interpreted with great caution due to the sample size. Further, we conducted a pharmacist-led medication review to evaluate whether patients were prescribed medication affecting hydration. The pharmacist identified that 60% of the study sample were prescribed medication that may cause dehydration, and 52% had been prescribed medication that may cause overhydration. One-third of the study sample had both types of medication. The findings above may suggest that muscle mass estimates might be biased by hydration status. Therefore, future work should conduct an in-depth evaluation of how hydration status may affect the validity of muscle mass estimates in a larger sample of older patients. 

Our study sample consisted of a limited number of patients with low muscle mass according to GLIM and EWGSOP-2 definitions (10–31%). However, we observed a high prevalence of malnutrition and risk of malnutrition (68%), which may contradict the assumption that malnutrition and low muscle mass are closely intercorrelated [3]. The prevalence of malnutrition and risk of malnutrition in this study is comparable to the prevalence reported in the literature [6,37]. Like the general assumption, we observed that patients being malnourished or at risk of malnutrition had lower absolute muscle mass estimates compared to well-nourished patients. Further, when applying ASM estimated with MF-BIA, more patients were identified with low muscle mass compared to the height-adjusted ASMI variable, indicating that the prevalence of low muscle mass is dependent on the applied muscle mass variable. This tendency was also observed in the study by Sousa-Santo et al. [33]. The rather dense distribution of patients near the ASM cut-off values, as seen in Figure 3, may contribute to uncertainties about the validity of current cut-off values. We could observe that for multiple cases of discrepancies, the values were quite close to the cut-off points. The four patients, with an ASM value of a maximum of 1.5 kg more than current ASM cut-off values, were malnourished or at risk of malnutrition, and three had poor appetite. These findings raise the question about the most appropriate muscle mass variable and the validity of current cut-off point to identify low muscle mass in older patients. Therefore, we believe further prospective studies evaluating which cut-off point is best at identifying adverse clinical outcomes are urgently needed.

### 4.3. Strengths and Limitations

A strength of our study is that MF-BIA and DXA measurements were performed by trained personnel on the same day immediately after each other. Another strength of our study is the comparison of three different muscle mass estimates within hydration groups since abnormal hydration status may contribute to measurement errors [10].

Some study limitations are important to mention: Firstly, the sample size was limited and based on specific inclusion and exclusion criteria from the main study, potentially compromising external validation. Furthermore, potential selection bias may have been introduced as some patients declined to participate without providing a reason, and a skewed sex distribution was observed. In total, 36% of our sample were male. As the study is exploratory and not based on a sample size calculation, it can be challenging to interpret the significance of the study results. Wide CI was observed for sensitivity, specificity, PPV, and NPV, reflecting a large uncertainty of the estimates. Therefore, these results need to be interpreted with caution. Another limitation of our study is the lack of a strict standardization protocol. However, we aimed to design a feasible study reflecting clinical practice where strict standardization protocols are not always possible, e.g., in busy patient wards such as an ED and due to ethical aspects of demanding fasting in malnourished patients. A systematic review of seven studies evaluating the utility of BIA in the diagnosis of sarcopenia reports that only one study had described a fasting protocol [38]. However, Lozano-Nieto and Turner and Gibson et al. have shown that orthostatic fluid shifts may bias the body composition estimates in healthy younger male adults [39,40]. 

In this study, the gold standards for non-invasive estimates of muscle mass (Magnetic Resonance Imaging (MRI) and computed tomography scans) were not performed. Instead, we used the DXA-based approach as a reference method [2]. DXA holds methodological problems and uncertainties. Firstly, studies show substantial variation in muscle mass estimates when comparing different DXA devices, software, or different models from the same manufacturer [41]. Secondly, the major concern with the estimation of muscle mass with DXA is the influence of abnormal hydration, which is highly prevalent in older adults [2,41,42]. Two studies found that, in older adults, DXA estimated total muscle mass higher than creatinine-based measurements and whole-body MRI scans and concluded that extracellular fluid accumulation may be an important contributor to this inconsistency [43,44]. Together, the errors of measurements in this study might have influenced both MF-BIA and DXA. 

## 5. Conclusions

In this exploratory study, our results indicate that the estimation of absolute values of muscle mass with MF-BIA in older hospitalized patients should be interpreted with caution and that the two techniques cannot be applied interchangeably. MF-BIA appeared reasonable for identifying low muscle mass, taking its feasibility into consideration, but future studies with larger study samples are urgently needed to provide more precise and reliable muscle mass estimates.

Researchers should carefully interpret results from both BIA and DXA in the diagnosis of malnutrition and sarcopenia. Indeed, inappropriate measures can lead to over- or underestimation of low muscle mass, ultimately affecting the accuracy in the diagnostics of malnutrition and sarcopenia in older hospitalized patients. 

## Figures and Tables

**Table 1:** Muscle mass variables and cut-off points for low muscle mass as recommended by GLIM and EWGSOP-2.

Muscle Mass Variable	Cut-Off Points Female	Cut-Off Points Male
Appendicular skeletal muscle mass (ASM, kg) ^1^	<15 kg	<20 kg
Appendicular skeletal muscle mass index (ASMI, kg/m^2^) ^1^	<5.5 kg/m^2^	<7 kg/m^2^
Fat-free mass index (FFMI, kg/m^2^) ^2^	<15 kg/m^2^	<17 kg/m^2^

^1^ GLIM and EWGSOP-2 recommendations. ^2^ GLIM recommendation only.

**Table 2:** Patient characteristics.

	Total (n = 42)	Female (n = 27)	Male (n = 15)
**Variable**			
Age, years, mean (SD)	78.9 (6.8)	79.9 (6.7)	76.9 (6.5)
Weight, kg, mean (SD)	75.8 (20.3)	70.7 (20.7)	85.1 (16.4)
Height, cm, mean (SD)	167.6 (7.8)	163.8 (6.6)	174.3 (4.7)
BMI, mean (SD)	27.0 (6.4)	26.4 (7.0)	28.2 (5.0)
Admission diagnosis category ^1^			
Cardiovascular, n (%)	13 (31%)	7 (26%)	6 (40%)
Gastrointestinal, n (%)	3 (7%)	3 (11%)	0 (0%)
Infectious, n (%)	11 (26%)	7 (26%)	4 (27%)
Lab abnormality, n (%)	1 (2%)	1 (4%)	0 (0%)
Mechanical, n (%)	3 (7%)	1 (4%)	2 (13%)
Nervous, n (%)	5 (12%)	4 (15%)	1 (7%)
Respiratory, n (%)	5 (12%)	3 (11%)	2 (13%)
Social, n (%)	1 (2%)	1 (4%)	0 (0%)
CCI = 0 ^2^	25 (63%)	18 (72%)	7 (47%)
CCI = 1	11 (28%)	5 (20%)	6 (40%)
CCI = 2	4 (10%)	2 (8%)	2 (13%)
LOS, days, median (IQR) ^3^	1.50 (0.00:3.25)	0.50 (0.00:2.75)	3.00 (0.25:4.00)
Readmission, 30 days, n (%)	31 (74%)	20 (74%)	11 (73%)
Mortality, 3 months, n (%)	3 (7%)	1 (4%)	2 (13%)
Mortality, 1 year, n (%)	3 (7%)	1 (4%)	2 (13%)
MNA^®^-SF score 12–14, n (%) ^4^	13 (32%)	9 (33%)	4 (29%)
MNA^®^-SF score 8–11, n (%)	19 (46%)	13 (48%)	6 (41%)
MNA^®^-SF score 0–7, n (%)	9 (22%)	5 (19%)	4 (29%)
SNAQ score > 15, n (%)	22 (52%)	15 (56%)	7 (47%)
SNAQ score ≤ 14, n (%)	20 (48%)	12 (44%)	8 (53%)
Dehydration > 295 mOsm/L, n (%) ^5^	12 (32%)	6 (23%)	6 (50%)
Medication, dehydrating effect, n (%) ^6^	25 (60%)	16 (59%)	9 (60%)
Medication, overhydrating effect, n (%) ^7^	22 (52%)	12 (44%)	10 (67%)

Abbreviations: SD: standard deviation, BMI: body mass index, CCI: Charlson Comorbidity Index, LOS: length of stay, IQR: interquartile range, MNA^®^-SF: Mini Nutritional Assessment Short-Form, SNAQ: Simplified Nutritional Appetite Questionnaire. ^1^ Patients can have >1 admission diagnosis, ^2^ CCI values are missing for two patients, ^3^ LOS value is missing for one patient, ^4^ MNA^®^-SF values are missing for two patients, ^5^ dehydration values are missing for four patients, ^6,7^ patients may be prescribed >1 medications with dehydrating and/or overhydrating effect.

**Table 3:** Absolute values of muscle mass and prevalence of low muscle mass according to MF-BIA and DXA.

		All Patients (n = 42)		
		ASM (kg)	ASMI (kg/m^2^)	FFMI (kg/m^2^)
**Absolute values of muscle mass**				
DXA, mean (SD)		20.5 (5.5)	7.3 (1.6)	17.3 (2.6)
MF-BIA, mean (SD)		21.2 (5.4)	7.5 (1.4)	18.0 (2.3)
**Prevalence of low muscle mass**				
DXA, n (%)		7 (17%)	9 (21%)	13 (31%)
MF-BIA, n (%)		8 (19%)	3 (7%)	4 (10%)

Abbreviations: ASM: appendicular skeletal muscle mass, ASMI: appendicular skeletal muscle mass index, FFMI: fat-free mass index, DXA: dual-energy X-ray absorptiometry, SD: standard deviation, MF-BIA: multi-frequency bioelectrical impedance analysis.

**Table 4:** Evaluation of MF-BIA against DXA to estimate absolute values of muscle mass and low muscle mass.

	All Patients (n = 42)		
	ASM (kg)	ASMI (kg/m^2^)	FFMI (kg/m^2^)
Mean bias, mean (CI) ^1^	0.63 (−0.20:1.46)	0.21 (−0.08:0.50)	0.76 (0.31:1.22)
Mean bias, % (CI)	3.9 (0.2:7.6)	3.9 (0.2:7.6)	5.1 (2.4:7.8)
LOA, lower, upper	−4.61, 5.87	−1.62, 2.04	−2.08, 3.61
5% accurate estimations, % (CI)	33 (20:50)	33 (20:50)	48 (32:63)
10% accurate estimations, % (CI)	62 (46:76)	62 (46:76)	74 (58:86)
15% accurate estimations, % (CI)	79 (63:89)	79 (63:89)	88 (74:96)
Sensitivity, (CI)	0.86 (0.42:1.00)	0.22 (0.03:0.60)	0.23 (0.05:0.54)
Specificity, (CI)	0.94 (0.81:0.99)	0.97 (0.84:1.00)	0.97 (0.82:1.00)
PPV, (CI)	0.75 (0.35:0.97)	0.67 (0.09:0.99)	0.75 (0.19:0.99)
NPV, (CI)	0.97 (0.85:1.00)	0.82 (0.67:0.92)	0.74 (0.57:0.87)
Kappa, (CI)	0.76 (0.46:1.06)	0.25 (0.00:0.50)	0.24 (0.01:0.48)
Agreement, % (Cl)	93 (79:98)	81 (65:91)	74 (58:86)

Abbreviations: CI: confidence intervals, ASM: appendicular skeletal muscle mass, ASMI: appendicular skeletal muscle mass index, FFMI: fat-free mass index, LOA: limits of agreement, PPV: positive predictive value, NPV: negative predictive value. ^1^ Bias is defined as mean values of MF-BIA—DXA.

## Data Availability

Data from this study are available on request from the corresponding author. Data are not publicly available due to regulations set out by the Danish Data Protection Agency.

## Supplementary Materials

The following supporting information can be downloaded at: https://www.mdpi.com/article/10.3390/jcm13010196/s1, Tables S1 and S2: list of prescribed medication belonging to the selected medication classes.
