# Supplementary material for: Evaluation of Multi-Frequency Bioelectrical Impedance Analysis against Dual-Energy X-ray Absorptiometry for Estimation of Low Muscle Mass in Older Hospitalized Patients
Source: J Clin Med. 2023 Dec 29;13(1):196. doi: 10.3390/jcm13010196 (PMC10779600; doi:10.3390/jcm13010196)
Supplement: Supplementary file 1 [file jcm-13-00196-s001.zip › jcm-2748182-supplementary.pdf]

Supplementary Material

Table S1. Medication classes.

	**Medication classes with dehydrating effect including ATC**
**Loop diuretics**	Furosemid (C03CA01)
Thiazides	Bendroflumethiazid (C03AB01); Hydrochlorthiazid (C03AA03)
Potassium-sparing diuretics	Amilorid, furosemid (C03EB01)
RAAS-inhibitors	Candesartan (C09CA06); Enalapril (C09AA02); Losartan (C09CA01); Ramipril (C09AA05); Valsartan (C09CA03)
	**Medication classes with overhydrating effect including ATC**
Calcium-antagonists	Amlodipin (C08CA01); Felodipin (C08CA02); Lercanidipin (C08CA13); Nifedipin (C08CA05); Verapamil (C08DA01)
Alpha- and beta-blockers	Carvedilol (C07AG02); Metoprolol (C07AB02)
NSAIDs	Ibuprofen (M01AE01)
glucocorticoids	Prednisolon (H02AB06)
Urinary tract agents	Alfuzosin (G04CA01); Mirabegron (G04BD12); Tamsulosin (G04CA02)

Table S2. Medication related to hydration.

	**Total (=42)**	**Female (n=27)**	**Male (n=15)**
Medication dehydrating effect, n (%)	25 (60%)	16 (59%)	9 (60%)
Loop diuretics, n (%)	16 (38%)	10 (37%)	6 (40%)
Thiazides, n (%)	7 (17%)	4 (15%)	3 (20%)
Potassium-sparing diuretics, n (%)	1 (2%)	1 (4%)	0 (0%)
RAAS-inhibitors, n (%)	17 (41%)	11 (41%)	6 (40%)
Medication overhydrating effect, n (%)	22 (52%)	12 (44%)	10 (67%)
Calcium-antagonists, n (%)	11 (26%)	6 (22%)	5 (33%)
Alpha- and beta-blockers, n (%)	11 (26%)	5 (19%)	6 (40%)
NSAIDs, n (%)	4 (10%)	3 (11%)	1 (7%)
Glucocorticoids, n (%)	2 (5%)	1 (4%)	1 (7%)
Urinary tract agents, n (%)	5 (12%)	2 (7%)	3 (20%)
